# Community-based management of a five-arm randomised clinical trial in COVID-19 outpatients in South Africa: challenges and opportunities

**DOI:** 10.1186/s13063-023-07577-6

**Published:** 2023-10-04

**Authors:** Nomathemba Chandiwana, Chelsea Kruger, Naomi Richardson, Sibongiseni Nxumalo, Nkoleleng Mashilo, Yengiwe Dineka, Ntanganedzeni Mudau, Hilary Johnstone, Wookyung Kim, Chung Ju, Sarah Arbe-Barnes, Anne Claire Marrast, Julia Flynn, Willem D. Francois Venter

**Affiliations:** 1 Ezintsha, Faculty of Health Sciences, University of the Witwatersrand, Building C, 32 Princess of Wales Terrace, Johannesburg, 2001 South Africa; 2 Magenta Communications Ltd, Abingdon, Oxfordshire UK; 3 HJ-Clinical Trial Consultancy, George, South Africa; 4 Shin Poong Pharm. Co. Ltd, Seoul, Republic of Korea; 5 Graduate School of Clinical Pharmacy, CHA University, Pocheon-Si, Gyeonggi-Do, Republic of Korea; 6 Artemida Pharma, Stevenage, UK; 7 Medicines for Malaria Venture, Geneva, Switzerland

**Keywords:** COVID-19, Clinical research management, South Africa, Drug repurposing, Outpatient, Clinical trial design

## Abstract

**Background:**

Repeated COVID-19 waves and corresponding mitigation measures have impacted health systems globally with exceptional challenges. In response to the pandemic, researchers, regulators, and funders rapidly pivoted to COVID-19 research activities. However, many clinical drug studies were not completed, due to often complex and rapidly evolving research conditions.

**Methods:**

We outline our experience of planning and managing a randomised, adaptive, open-label, phase 2 clinical trial to evaluate the safety and efficacy of four repurposed drug regimens versus standard-of-care (SOC) in outpatients with ‘mild to moderate’ COVID-19 in Johannesburg, South Africa, in the context of a partnership with multiple stakeholders. The study was conducted between 3 September 2020 and 23 August 2021 during changing COVID-19 restrictions, significant morbidity and mortality waves, and allied supply line, economic, and political instability.

**Results:**

Our clinical study design was pragmatic, including low-risk patients who were treated open label. There was built-in flexibility, including provision for some sample size adjustment and a range of secondary efficacy outcomes. Barriers to recruitment included the timing of waves, staff shortages due to illness, late presentation of patients, COVID-19 misinformation, and political unrest. Mitigations were the use of community health workers, deployment of mobile clinical units, and simplification of screening. Trial management required a radical reorganisation of logistics and processes to accommodate COVID-19 restrictions. These included the delivery of staff training and monitoring remotely, electronic consent, patient training and support to collect samples and report data at home, and the introduction of tele-medicine. These measures were successful for data collection, safe, and well received by patients.

**Conclusion:**

Completing a COVID-19 trial in outpatients during the height of the pandemic required multiple innovations in nearly every aspect of clinical trial management, a high commitment level from study staff and patients, and support from study sponsors. Our experience has generated a more robust clinical research infrastructure, building in efficiencies to clinical trial management beyond the pandemic.

## Background

Reported as a novel human pathogen in December 2019 in Wuhan, China [1], SARS-CoV-2 spread rapidly, with coronavirus disease (COVID-19) designated a pandemic by the World Health Organization on March 11, 2020 [2]. Nearly 3 years into the pandemic, there were at least 15 million deaths directly or indirectly attributable to COVID-19 [3], and the pandemic had impacted every aspect of life.

The global research community mobilised to address the emergency. Vaccine development had an uncertain timescale and success was far from guaranteed [4–6]. Later, it became evident that vaccine access would not be equitable [7]. Repurposing affordable drugs appeared to offer a rapid route to treatment as the pharmacokinetics, safety, and tolerability of candidates were already well described and clinical trials in COVID-19 could be swiftly implemented [8–11].

African countries were thought to be vulnerable to COVID-19, with overcrowding in urban areas, limited access to sanitation and water, fragile health systems, and a high infectious disease burden [12–16]. In March 2020, the African Academy of Sciences initiated a research and development priority setting exercise with 276 African scientists [17]. Clinical trials were identified as a key priority to establish the feasibility, safety, and efficacy of vaccines and drugs for COVID-19 in Africa [17].

In Africa, without a vaccine and limited access to hospital treatment, the urgent need was for affordable COVID-19 drugs that could be given safely in the community at symptom onset to reduce the risk of disease progression, hospitalisation, and death [18]. To achieve these aims, clinical trials of repurposed drug candidates in African populations was a priority. Focusing on repurposed drugs, which are existing medications that are being tested for new therapeutic uses, could offer a more accessible and timely solution for managing COVID-19 in Africa, where resources like COVID-19 vaccines and advanced hospital treatments may be scarce. By conducting trials within African populations, researchers can also gather data that is relevant to the local context and better understand the effectiveness and safety of these drugs in this specific demographic.

**Fig. 1:**
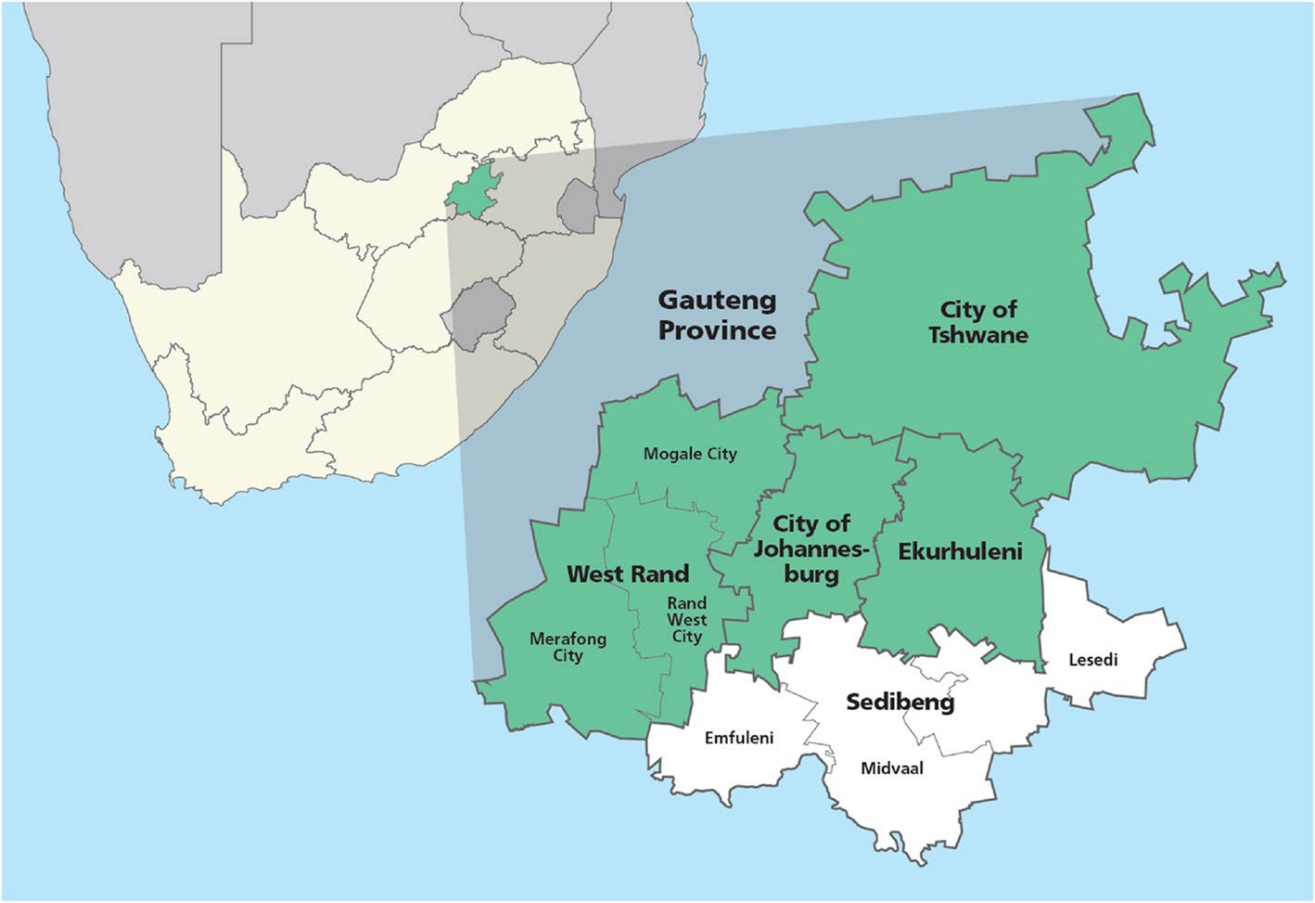
Location of the study area (shaded green) in Gauteng Province, South Africa. Redrawn and adapted from: File: Map of Gauteng with municipalities blank (2016).svg (https://commons.wikimedia.org/wiki/File:Map_of_Gauteng_with_municipalities_blank_(2016).svg)

**Fig. 2:**
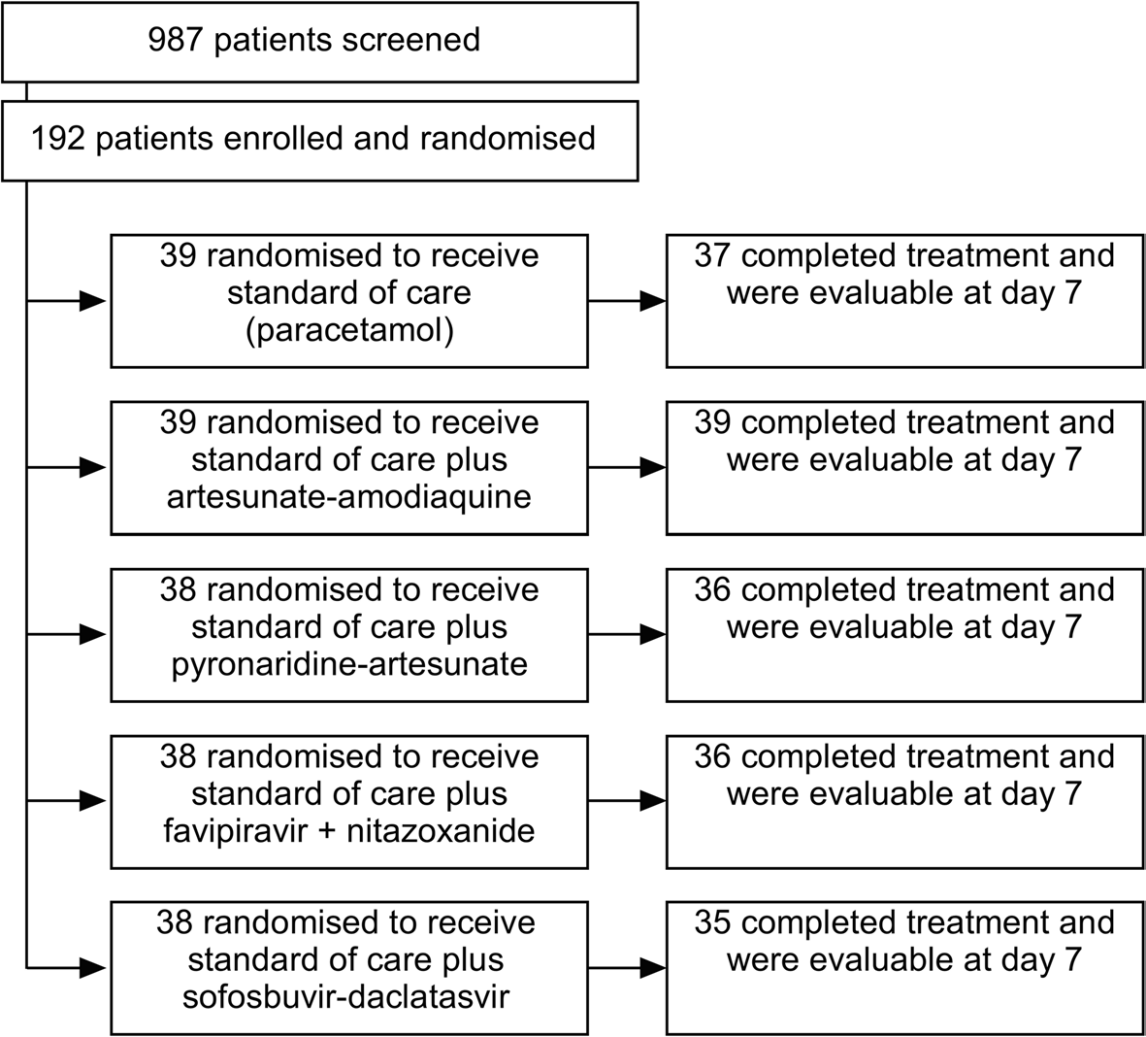
Study summary

In response to this urgent need, we conducted a randomised, open-label, phase 2 clinical trial to evaluate the safety and efficacy of four repurposed drug regimens versus standard-of-care (SOC) in outpatients with COVID-19 in Johannesburg, South Africa, between 3 September 2020 and 23 August 2021 [19] (Fig. 1). The SOC arm comprised of paracetamol administered as needed, which remained the primary treatment option available for outpatient COVID-19 care in the public sector of South Africa [20]. However, it should be noted that the private sector in South Africa and other regions offered greater accessibility to a broader range of treatment options. Based on the available evidence, four drug regimens were selected: the antimalarial drugs artesunate-amodiaquine and pyronaridine-artesunate [21, 22], the combination of the antiviral favipiravir [23] and antiparasitic nitazoxanide [24], and the fixed-dose combination sofosbuvir-daclatasvir, approved for the treatment of hepatitis C [25] (Fig. 2).

A search of ClinicalTrials.org (15 November 2022) reported that studies in outpatients represented just 6.5% (84/1,303) of all COVID-19 interventional studies started between Jan 1, 2020, and Dec 31, 2020. Less than half of these studies (35/84) had completed recruitment by November 2022. There were only two completed outpatient studies that recruited African patients; one investigated molnupiravir (NCT04575597) and the other was our study (NCT04532931) [19, 26]. A search of the Pan-African Clinical trials registry did not identify any additional completed outpatient drug trials that started in 2020 [27].

There has been wide-ranging dialogue around the impact of COVID-19 on healthcare delivery and staff [28, 29], pandemic preparedness [30–33], and the challenges of conducting or maintaining clinical trials across various therapeutic areas during the pandemic [34, 35]. There has also been some discussion around design and reporting issues for clinical trials on COVID-19 [36–38]. However, due to the limited studies and high attrition rates, the challenges, mitigations, and opportunities of conducting trials in outpatients with COVID-19 have not been well documented. Here, we discuss the planning and implementation of our five-arm clinical trial for the outpatient treatment of COVID-19 in South Africa against the background of multiple severe COVID-19 waves, economic and supply line instability, regulatory complexity, changes in endpoints, and severe political instability.

## Study planning

**Fig. 3:**
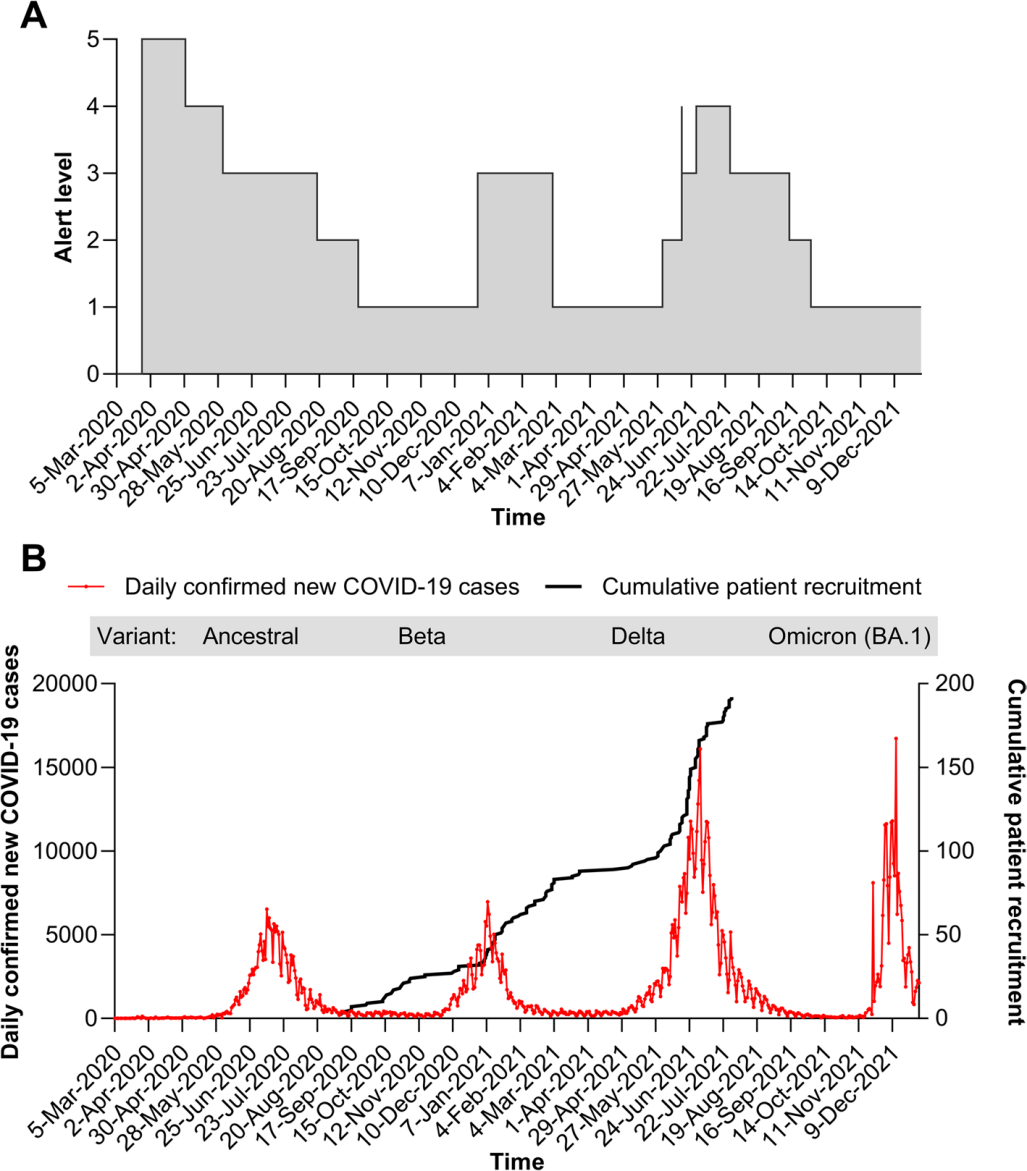
Timeline of the pandemic responses and trial recruitment. **A** Timing of COVID-19 alert levels across South Africa [39]. **B** Cumulative patient recruitment versus active COVID-19 cases in Gauteng province [40]

When the protocol was planned in February to May 2020, very little was known about COVID-19. The first wave was devastating Europe. South Africa had already seen pre-emptive lockdowns and was anticipating the worst regarding the clinical impact of the virus infection (Fig. 3A) [39, 40]. The protocol had to be achievable within the context of stringent lockdown and isolation restrictions, and a health system that was poorly prepared for a pandemic, while maximising the opportunities for identifying a successful treatment and ensuring patient safety. Given the anticipated barriers to recruitment within the community setting during COVID-19 restrictions, the target sample size had to be realistically achievable.

### Stakeholder collaboration

Study protocol development involved various stakeholders, including the investigators, funding bodies, study sponsors, the South African Health Products Authority (SAHPRA), and the Gauteng Provincial Government as well as review by a scientific advisory committee comprising investigators and academics involved in other COVID-19-related projects. The process of agreeing on the protocol was complicated by uncertainties around the most relevant endpoints and the need to carefully examine emerging evidence regarding the potential risks and benefits for the investigational drugs in a new disease in a patient population that might not otherwise have healthcare access, and so be vulnerable to undue influence [41–43].

The protocol was finalised in May 2020, with recruitment planned to begin in July 2020. However, further delays caused by repeated reviews of the protocol pushed this back to September, missing the first COVID-19 wave (Fig. 3B). Not only did this slow recruitment, but by September, 29.3% (53/181) of evaluable patients were positive for SARS CoV-2 serology at baseline, indicating prior exposure to the virus and undermining the ability to detect a drug effect [19]. The delays also contributed to a substantial increase in study costs due to extended staff contracts, administrative expenses, intensified patient recruitment efforts, and increased data management and monitoring requirements.

The challenges posed by delay/uncertainty with regard to recruitment made it necessary to ensure very frequent alignment and sharing of information between the donors, the sponsor, the operational lead, the clinical research organisation, and the study site. These requirements to regularly develop and update risk mitigation and contingency plans represented an additional burden to a complex project. Certain activities required approval from multiple stakeholders prior to implementation, sometimes resulting in significant delays. Also, some study drugs had to be resupplied after reaching their expiry date.

With multiple stakeholder involvement, clear and efficient boundaries need to be established to define responsibilities, reporting channels, and processes at study inception. This is especially important where new working relationships are being fostered. In addition, there must be upfront agreement to adhere to protocol review timelines with pre-determined decision points to avoid later delays, unless it is absolutely necessary to postpone decisions pending availability of further data. Given the uncertainty around conducting clinical trials in a new disease, a financial buffer to mitigate unanticipated expenses should also be accounted for at study start.

### Pragmatic design

The study design has been described in detail elsewhere [19]. In brief, this phase 2, exploratory, single-centre, open-label, randomised, adaptive clinical trial was conducted in South Africa to compare the effectiveness and safety of four repurposed anti-infective drug regimens in treating COVID-19 in outpatient settings versus SOC. Symptomatic outpatients aged ≥ 18 to ≤ 65 years, with confirmed SARS-CoV-2 infection, were randomly assigned to one of the following treatment groups: SOC paracetamol, SOC plus artesunate-amodiaquine, pyronaridine-artesunate, favipiravir plus nitazoxanide, or sofosbuvir-daclatasvir [19].

The ambition was to obtain a rapid efficacy readout for these promising drugs to address the healthcare emergency. Only favipiravir had been tested in COVID-19 patients in two hospital-based studies [44, 45]. Thus, the four investigational regimens were selected based on in vitro activity against SARS-CoV-2, physiologically based pharmacokinetic modelling, affordability, and safety consistent with community treatment [19]. For a conventional drug development process, proof-of-concept and dose-finding studies would be required. However, because we were investigating repurposed drugs, we used standard doses for the approved indications, as an approach to accelerate development [46].

An open-label design was necessary as matched placebos could not be sourced during a pandemic. Also, the high pill burden would increase complexity and undermine adherence. As our study was not designed to compare investigational arms but to compare each of the four parallel arms to a single SOC arm, and as there was an objective endpoint, there was little advantage to a blinded design [19].

A low-risk patient population was targeted, aged ≥ 18 to ≤ 65 years and with mild-to-moderate symptoms [19]. This was a pragmatic response to the potential risks and benefits for mostly untested therapies in a new disease, given the intense pressure on the health system and with all patients treated and followed up as outpatients.

An objective primary endpoint was needed to drive efficiency of sample size. Virological clearance at day 7 was considered a feasible endpoint. At the time of study initiation, our opinion was that reliable data could be obtained as community-based reverse transcription polymerase chain reaction (RT-PCR) testing for confirmation of SARS-CoV-2-positivity had been rapidly adopted and expanded in South Africa [47]. However, as there was uncertainty regarding the clinical relevance of day 7 virological clearance, various other virological and clinical secondary endpoints were included in the protocol as contingency [19].

Published evidence suggesting that 20% of patients would have viral clearance at day 7 in the SOC arm, increasing to 50% with effective drug therapy, was used for power and sample size calculation [44]. Based on these data, a sample size of 40 patients per arm was required for ≥ 80% power, i.e., a total of 250 patients assuming 20% loss to follow-up. This appeared an achievable target given the trajectory of the pandemic observed in Europe between February and May 2020. However, there was some flexibility in the protocol for a reassessment of recruitment targets based on the actual number of patients lost to follow-up.

### Insights on the design and recommendations

The study was underpowered for the primary endpoint as 34.2% (13/38) of patients in the SOC arm had virological clearance at day 7, much higher than the expected 20% [19]. Achieving a balance between sample size feasibility and the capacity to detect a treatment effect requires knowledge of the expected effect size and variability. In our study, the available information was meagre and our estimations imperfect. One approach could have been to calculate a range of plausible sample sizes and allow adjustment within these pre-determined scenarios based on interim analyses, though resources would need to be secured for all scenarios and interim analyses at inception. Study efficiency could be increased by using paired sampling to reduce variability, using a continuous variable (e.g., viral load), increasing the numbers of patients in the SOC arm, or restricting the population to patients with risk factors for the effect being measured [48]. After careful consideration, we decided not to include additional arms in the trial. One of the key reasons for not adding more arms was the current lack of a fully established predictive relationship between the magnitude and timing of viral RNA reduction and viral infectivity or clinical benefit at interim analysis. Including additional arms without a solid understanding of these correlations could potentially introduce confounding factors and complicate the interpretation of the results.

Although a patient population with higher risk may have increased the potential to detect differences in outcome between the treatment groups, this was not an option. Even though only two patients required hospitalisation during the study, one had to be treated at home with supplemental oxygen because of a lack of hospital beds and social unrest [19, 49]. This illustrates the insufficient capacity within the health system at that time to safely conduct a clinical trial in a population that was at heightened risk of disease progression.

In retrospect, having fewer virological outcomes would have simplified trial management and analysis. However, although the RT-PCR-driven primary endpoint seemed appropriate at the time of design, it later became evident that while this was a sensitive indication of initial infection it was a poor indicator for clearance of replicating virus. For new diseases, validated endpoints are likely unavailable and there is a balance between anticipating the most relevant endpoints and maintaining trial feasibility. Similarly, disease severity was evaluated using the WHO Ordinal Scale for Clinical Improvement [50], the FLU-PRO® Plus questionnaire, and FLU-PRO® Plus Global Additional Diary Items [51]. Conducting three surveys placed considerable demands on patients and staff. The WHO Ordinal Scale encompasses the wide spectrum of COVID-19 outcomes and includes mortality as a component but requires significant ongoing input from trained health professionals. The FLU-PRO surveys allow the assessment of sustained clinical recovery, which is a key consideration in populations with low mortality and hospitalisation rates. However, the capturing burden is high because of the daily assessment and the variety of changes in symptoms between different variants. Also the high rates of spontaneous recovery make it difficult to detect a significant difference between SOC and treatment for this outcome. The selection of limited, highly feasible, and readily obtained endpoints is recommended rather than diverting effort to endpoints that might be interesting or consistent with conventional drug efficacy trials, but which are difficult to secure during a health emergency. However, these decisions will likely be constrained by a lack of evidence.

Conducting a five-arm trial at a single site in a pandemic was ambitious. Additional trial sites would have supported recruitment and should be considered where infection incidence progresses in waves or is seasonal. In the early stages of the pandemic, a more compact three-arm study would have increased feasibility. An alternative approach is to adopt a platform trial approach where treatment options are tested, reviewed, and either progressed or replaced depending on the findings under a single protocol. These studies require considerable efforts to establish, an ongoing funding commitment, and usually involve multiple trial sites often across several countries [52]. However, the ANTICOV platform study, which commenced recruiting COVID-19 outpatients in April 2021, demonstrates that such designs are feasible for the evaluation of community-based treatments in Africa and other low-resource settings [53].

## Study management

### COVID-19 restrictions

Conducted at the height of the pandemic, 687,511 COVID-19 cases and 14,696 deaths were recorded across Gauteng province over the study duration [40]. Trial management was dominated by Government-imposed COVID-19 restrictions, with little warning of changes (Fig. 3A). Travel, large gatherings, work/school attendance, and alcohol and tobacco were restricted, with face masks and hygiene measures introduced. Controls were vigorously enforced, particularly early in the pandemic, causing significant hardship to poorer communities [54–57].

In the context of COVID-19, “alert levels” refer to a system used by governments or health authorities to categorise the severity of the COVID-19 pandemic and guide public health responses. In South Africa, five alert levels were defined, though specific restrictions varied within each level based on experience and what was politically acceptable (Table 1) [39]. Our study was planned during a national disaster, with stringent restrictions on leaving the home (Fig. 3A). Although restrictions eased and then tightened, the operational organisation of the study was designed to be resilient to the evolving environment. This approach also enhanced the confidence of study staff to effectively fulfil their roles when alert levels changed and was a key element in ensuring trial completion (Table 2).

**Table 1:** Summary of COVID-19 alert levels in South Africa [39]

Alert level	Criteria	Objective
5	High COVID-19 spread with a low health system readiness	Drastic measures to contain the spread of the virus and save lives
4	Moderate to a high COVID-19 spread with a low to moderate health system readiness	Extreme precautions to limit community transmission and outbreaks, while allowing some activity to resume
3	Moderate COVID-19 spread with a moderate health system readiness	Restrictions on many activities, including at workplaces and socially, to address a high risk of transmission
2	Moderate COVID-19 spread with a high health system readiness	Physical distancing and restrictions on leisure and social activities to prevent a resurgence of the virus
1	Low COVID-19 spread with a high health system readiness	Most normal activity can resume, with precautions and health guidelines followed at all times. Population prepared for an increase in alert levels if necessary

**Table 2:** Operational impact of COVID-19 on clinical trials conduct

Area of consideration	Adaptation for COVID-19 response
Clinical site infrastructure	• Existing workspaces and shared office zones enlarged to accommodate social distancing • Physical barriers set up between colleagues and patients • Separate isolation areas with a new entrance built to accommodate patients under investigation • Additional staff required, or current staff took on an additional role, for screening procedures at entrance • PPE was procured urgently for all staff • Staff training amidst rapidly evolving guidelines was challenging, especially for non-clinical team members
Logistical operations of staff	• Taxi companies contracted to ensure staff could safely get to site during lockdown • Hybrid work arrangements were implemented • Site initiation visits and meetings were held virtually
Clinical trial set up and approvals	• Regulatory authorities and ethical body approval process were expedited with electronic submissions • Use of e-consent procedures
Recruitment, retention and follow ups	• Community healthcare worker engagement became intensive and indispensable to drive recruitment and ensure retention • New logistical consideration of providing transport for those reliant on public transport translated to hiring drivers and rental cars • Fully equipped mobile vans were deployed into communities to minimise the number of patients being seen on site • Introduction of telemedicine and trial-specific smart phone apps, which is unfamiliar for many South African healthcare workers who are accustomed to in-person consultations • Training and support for patients on completing and reporting study assessments from home
Monitoring and data management	• Remote monitoring became more prevalent • On-site monitoring visits were often postponed, leading to the possibility of delayed recognition of areas requiring improvement
Patient education	• Counselling patients on health and hygiene habits, as well as addressing fear, stigmatisation, media misinformation and vaccine hesitancy, was actively incorporated into each point of contact

### Challenges and opportunities for patient recruitment

Patient recruitment was initiated during a lull in COVID-19 infections and progressed very slowly at first (Fig. 3B) [40]. In early September 2020, COVID-19 screening and testing activities at hospitals and primary care clinics across the province had stopped and quarantine sites were closed. Testing patterns also changed; public sector hospitals only tested patients with moderate-to-severe symptoms. Private labs had around 25 walk-in patients a day compared to 300 at the height of the first wave. Although ethical approval was granted from the University of the Witwatersrand, there were delays in obtaining approval from the Gauteng Provincial Department of Health permitting recruitment from public clinics. Consequently, for the first 6 weeks of the study (3 September until 8 October 2020), patients could only be recruited via referrals from nearby healthcare facilities. Even once recruiting from the community was possible, identifying patients within the enrolment window (symptoms starting ≤ 72 h prior to randomisation) was challenging. Therefore, the protocol was amended in November 2020 to include patients with symptoms starting ≤ 96 h prior to randomisation. This increased the number of successfully screened patients but was still within the window for RNA detection in symptomatic patients so trial integrity was maintained.

During the second wave (December 2020 to February 2021), new methods of working were implemented and the extreme pressure on the health service, including staff sickness absences, plus a break for the Christmas holiday, reduced the capacity to enrol patients. Notably, there was a shift in the presenting symptoms of COVID-19-infected patients, with more complaining of predominantly gastrointestinal symptoms as opposed to respiratory symptoms. This was likely due to the emergence and spread of the Beta variant (B.1.351; also called GH/501Y.V2), first detected in South Africa in December 2020 [58].

Between January and April 2021 recruitment was mostly steady, despite a declining incidence of infection, as staff became accustomed to the new working methods, more staff were employed and trained, and staff sickness absences decreased (Fig. 3B). However, there was some disruption in February when Gauteng Province experienced unusually heavy rains and flooding. As most of the population is reliant on public transport or are pedestrians, this limited access to health facilities for testing. Few patients were enrolled from April to June 2021 as infection incidence was low.

There was a high proportion of negative tests in the study, with a SARS-CoV-2 positivity rate of 2% in April 2021. This was slightly below the national average of approximately 4–5%, possibly because our screening population was more diverse, so less likely to have COVID-19, or it could indicate a higher than average false-negative rate, which could be due to inadequate specimen collection or sampling too early in the course of infection. There were ongoing efforts to optimise the number of procedures at screening, which improved screening capacity. For example, split screening was implemented to reduce time spent on the main study consenting procedures in participants who subsequently tested negative for SARS-CoV-2 on PCR. A protocol amendment to allow saliva specimens (November 2020) did not yield the expected increase in recruitment. Other than a negative COVID-19 test, common reasons for screen failure were lack of highly effective contraception for female patients, concomitant use of efavirenz-based antiretroviral therapy, and unwillingness to participate in the study.

As pandemic fatigue set in, people became more complacent about regulations, and less likely to present to health facilities when developing mild symptoms, probably because of the negative economic consequences of self-isolation [59, 60]. The fracturing of social networks inhibited conventional routes for health communication, with a shift from receiving health education from healthcare providers and facilities to various and conflicting channels, such as billboards, text messages, app alerts, presidential addresses, press releases, and particularly social media. This increased the potential for misinformation to propagate and gain traction, including misconceptions of being protected against the virus or having low risk, and stigmatising responses to COVID-19 and medical mistrust [61, 62].

South Africa has an established network of community health workers (CHWs) supporting people living with HIV and contact tracing for tuberculosis. Nationally, 28,000 CHWs were re-deployed to conduct COVID-19 screening and contact tracing [54], with mobile COVID-19 units introduced to expand testing capacity. COVID-19 data were captured via a mobile phone app (COVID-Connect) and integrated into the existing influenza and pneumonia surveillance system allowing hot spots of infection to be targeted for recruitment. The influence of the CHWs who were experienced in going door-to-door in vulnerable communities was a critical component in reaching patients and countering misinformation.

During the winter (May to August 2021), there was a double burden of COVID-19 and influenza, both presenting with similar symptoms. With the third wave worsening throughout June and July, recruitment was at its highest rate. However, widespread civil unrest (9–18 July) with violence and looting, closure of services and businesses, and disruption to transport, food, fuel, and medical supplies necessitated a pause in recruitment. Recruitment was concluded on 29 July 2021 at 192 patients because of the lower than anticipated drop-out rate (Fig. 3B).

### Securing and supporting human resources

The pandemic progressed in a series of waves and the active use of epidemiological data was a limited tool to predict the timing and duration of future COVID waves. Staff were stood down between waves and had to be re-trained and re-deployed as case incidence increased, with the result that at the peak of each wave there were not enough trained staff to fully realise study enrolment potential.

Existing workspaces and shared office zones had to be enlarged to accommodate social distancing and physical barriers were set up between colleagues. Despite infection control measures within the trial and wider measures instituted by the government, staff were susceptible to COVID-19 infection and illness absences compounded staff shortages, particularly during the second wave. Study staff also had competing duties in the pandemic response.

Staff recruitment had to be done online and building a cohesive research team from people who had never met in person required innovative approaches, such as online group meetings where insights and issues could be shared. It was important that staff did not feel isolated and unsupported, particularly given the psychological pressure of stress, exhaustion, and separation from family and social support networks resulting from the pandemic response [63].

South Africa is confronted by HIV, tuberculosis, and chronic disease such as type 2 diabetes and hypertension [64–66]. Mobilisation of the expertise and resources applied to addressing these common infectious diseases enabled staff to quickly adapt to the requirements of running a COVID-19 trial. The core research team were experienced in conducting clinical trials in HIV and tuberculosis in vulnerable populations. Healthcare staff were skilled in advising patients on infection control and self-medication and were aware of the challenges facing patients who are not only unwell, but facing financial hardship, stigma, isolation, and the fear of an uncertain outcome [67].

Training staff amidst the rapidly evolving COVID-19 guidelines was complex, especially for non-clinical team members. The maintenance of a core team of trial staff that worked throughout the study was a key element in ensuring continuity of procedures, rapid pivoting when COVID-19 restrictions changed, and the delivery of high-quality training to peripheral staff. Multi-modal training was delivered remotely and in person, with materials such as presentations, operating procedures, and videos available online. Documentation, monitoring, and evaluation requirements were more demanding during the pandemic, and there were delays in monitoring that were unresolved by remote methods. However, there is scope to further streamline processes and to apply this approach for recruiting and training research teams in more normal circumstances.

### Supply chain management

Pandemic preparedness was inadequate in South Africa [68, 69], and the clinical trial commodity requirements were secondary to securing basic healthcare services. Drugs for this study were provided by the sponsor, with stocks sufficient for the entire study available at study start. More problematic was obtaining the necessary supplies of personal protective equipment (PPE). Global demand placed these commodities at a premium and quality assured sources were limited, with an influx of illegal and unstandardised distributors. Future pandemic preparedness could consider supporting the establishment of local accredited manufacturers and maintaining a stock of PPE reserved for the conduct of clinical trials. COVID-19 test kits were also in short supply but were procured directly for use in the clinical trial so as not to divert supplies from the pandemic response.

### Study procedures

Strict lockdown regulations and the need to protect staff and patients required radical and innovative measures. Infrastructure had to be modified, for example, separate isolation areas with a new entrance were built to accommodate patients under investigation for COVID-19. Screening and enrolment procedures were transferred from the study site to fully equipped clinic vans that could travel into communities, minimising the number of symptomatic patients seen on site. Concurrent clinical trials including immunocompromised patients were being conducted at the participating health facilities, so ensuring separation of suspected COVID-19 cases was imperative. Study personnel wore PPE during all interactions, and patients and study personnel were instructed to adhere to COVID-19-related safety measures, and the need to quarantine as per the current guidance.

Patients provided consent electronically, which was a new approach to use technology to address ethical obligations given the limits on in-person consultation. The process was compliant with regulatory requirements and provided seamless documentation through the enrolment process, allowing access to consent status while protecting patient’s personalised data.

A key innovation was that patients were trained and supported to conduct follow-up assessments independently by providing them with a password-protected and secure data collection tool, installed on their phones. This allowed participants to log in with details unique to them and perform self-assessments, including vital signs, oxygen saturation, and health surveys, minimising the need for travel and clinic visits. Where necessary, COVID-compliant transport to the clinic was provided, and patients were loaned the tools to complete online assessments. However, there were some issues in collecting information as patients did not always have sufficient electronic data capacity to upload the results.

Telemedicine is rarely available in African countries [70], but was successfully implemented in this study. Patients appreciated the convenience, and daily assessments would have been otherwise unachievable. This was a major cultural transition for clinical staff who were used to seeing patients in person. However, supporting patients conducting daily assessments provided extensive opportunities for interaction and the assessment of patients’ needs. This patient-centric approach is widely applicable [41], and acceptance was reflected in the positive feedback from patients and a high completion rate; only two patients withdrew from the study and three were lost to follow-up [19].

The package of risk mitigation strategies was shown to be robust during civil unrest in July 2021. Although recruitment was paused, other study activities progressed fluidly while ensuring patients and research staff were safe. Although necessary in the context of the pandemic, such adaptations can be applied to any trial where reducing the need for clinic visits would be safe and more convenient for patients. Extensions of this approach include patients receiving automatic notifications for follow up visits, the use of apps for patients to collect data, and an online booking system for patients to book follow-up visits or transport, etc. Such measures would further reduce the burden on clinical trial staff and provide patients with additional flexibility, control, and agency.

## Conclusions

As was the case for all trials initiated early in the pandemic, our study was designed based on an incomplete understanding of the disease, scant clinical evidence, and an evolving viral pathophysiology [36, 71]. Trial management not only had to address the logistical and operational issues of restrictions on movement and limited supplies, but also the psychological aspects for patients and staff, including the fear of infection, food and income insecurity, the breaking of social bonds, personal loss, and the deepening of social inequalities [55, 72]. Compromises had to be made, but also necessary innovations that have transformed the way we conduct clinical trials. Key elements in completing the trial were patients’ commitment to self-management and data gathering, the flexibility of study staff in adopting new methods of working, and the instigation of streamlined, resilient processes.

The pandemic has highlighted the need for a more robust, global clinical research infrastructure, agile enough to withstand unexpected emergencies. COVID-19 trials have demonstrated researchers’ adaptability and innovative thinking and the capacity to rapidly introduce a range of innovations. These have evolved to establish more modern and efficient processes for conducting all clinical trials. Also, applying these measures more broadly will protect clinical research efforts during future pandemics.

## Data Availability

This clinical trial is registered at ClinicalTrials.gov with the identifier NCT04532931 and the methods and results and conclusions are published [19]. De-identified patient data are available on reasonable request and with completion of a signed data access agreement from (https://www.mmv.org/about-us/contact-us) referencing this publication. Data will be available for at least 5 years from publication of this study.

## Abbreviations

COVID-19: Coronavirus disease of 2019
HIV: Human immunodeficiency virus
RT-PCR: Reverse transcription polymerase chain reaction
